# Psychosocial stressors and depression at a Swedish primary health care centre. A gender perspective study

**DOI:** 10.1186/1471-2296-12-120

**Published:** 2011-11-02

**Authors:** Ranja Strömberg, Lars G Backlund, Monica Löfvander

**Affiliations:** 1 Center for Family and Community Medicine, Department of Neurobiology, Caring Sciences and Society, Karolinska Institutet, Alfred Nobels allé 12, SE-12183 Huddinge, Sweden; 2 Center for Clinical Research Dalarna, Falun, Sweden

## Abstract

**Background:**

Psychosocial stress may account for the higher prevalence of depression in women and in individuals with a low educational background. The aim of this study was to analyse the association between depression and socio-demographic data, psychosocial stressors and lifestyle circumstances from a gender perspective in a relatively affluent primary care setting.

**Methods:**

Patients, aged 18- 75 years, visiting a drop-in clinic at a primary care health centre were screened with Beck's Depression Inventory (BDI). The physicians used also targeted screening with BDI. A questionnaire on socio-demographic data, psychosocial stressors and use of alcohol and tobacco was distributed. Among patients, who scored BDI ≥10, DSM-IV-criteria were used to diagnose depression. Of the 404 participants, 48 men and 76 women were diagnosed with depression. The reference group consisted of patients with BDI score <10, 187 men and 93 women. Age-adjusted odds ratios (ORs) with 95% confidence intervals (CI) as being depressed were calculated for the psychosocial stressors and lifestyle circumstances, separately for men and women. Multiple logistic regression analyses were used to determine the age-adjusted main effect models for men and women.

**Results:**

The same three psychosocial stressors: feeling very stressed, perceived poor physical health and being dissatisfied with one's family situation were associated with depression equally in men and women. The negative predictive values of the main effect models in men and women were 90.7% and 76.5%, respectively. Being dissatisfied with one's work situation had high ORs in both men and women. Unemployment and smoking were associated with depression in men only.

**Conclusions:**

Three questions, frequently asked by physicians, which involve patient's family and working situation as well as perceived stress and physical health, could be used as depression indicators in early detection of depression in men and women in primary health care.

## Background

Depression is a frequent disorder and thus also a common health problem to deal with in primary care [1-4]. The economic burden of depression in Sweden has doubled in the past decade owing to costs associated with reduced ability to work [5]. Worldwide, depression is one of the leading causes of ill health and disability, a trend that is likely to increase [6].

According to a meta-analysis, people with low socio-economic situation run a doubled risk of being persistently depressed but do not generally have a higher risk of new episodes [7]. A dose-response relation was observed for education and income, which provides compelling evidence for socio-economic inequality in depression [7]. Several studies have found psychosocial stressors such as unemployment, chronic social difficulties and persistent financial strains to be risk factors for depression [8-11].

The lifetime risk of depression is higher among women then men cross-culturally [12]. However, by integrating socio-economic variables, such as income and educational background, women's excess risk was reduced by approximately 50% [13]. Nonetheless, the reasons for gender differences in the prevalence of depression are not fully understood [14,15].

A study from primary health in Finland showed that adverse circumstances, such as traumatic life events, poor physical health, weakening interpersonal relationships, difficult socio-economic and work situations and alcohol problems are risk factors for depression and often occur simultaneously in some patients [16]. The same study showed that the associations between some of these risk factors and depression were stronger in men than women.

Managing patients with depression attending primary care should focus not only on medication for depression but also on psychosocial stressors. A Swedish study showed that patients with mental illness encounter a number of social problems [17], but no studies on risk factors specific to depression have been performed in Sweden.

In earlier studies we found that depression was common also in a relatively affluent area of Stockholm, Sweden [18,19]. The focus here was to explore risk factors for depression from a gender perspective to find depression indicators for physicians to use in clinical settings.

Therefore, the aims in this study were to analyse the association between depression and socio-demographic circumstances, psychosocial stressors and lifestyle choices in men and women living in a relatively affluent area in Sweden and asses any differences form a gender perspective.

## Methods

The study was conducted at a primary health care centre in central Stockholm, Sweden, that provided health care service for about 11.000 inhabitants. Almost all inhabitants were ethnic Swedes with a higher than the average educational background. Between 1997 and 2001, two of the five general practitioners working at the centre conducted the study during drop in clinic on week-days.

### Instruments and Questionnaire

To screen for depression, Beck's Depression Inventory (BDI) (cut-off score ≥10) was used [20,21]. DSM-IV - criteria were applied to diagnose depression [22] and the Montgomery-Åsberg Depression Rating Scale (MADRS) was used to measure severity of depression [23].

Participants were asked to complete the constructed Social Conditions (SC) questionnaire, which was based on literature [8,9,24]. It consisted of a series of questions with five fixed response alternatives designed to collect information on participants' socio-demographic data, psychosocial stressors and lifestyle choices. The questions are listed below as well as rating of the answers. The dichotomisation was done to identify patients with a high level of discomfort.

- How is your financial situation? The answers were dichotomised as poor (1) if the answer was 'very bad' or 'bad', and good (0) if the answer was 'neither bad nor good', 'good' or 'very good'.

- Time on sick leave during the past year was categorized as: ≥ 14 days (1) and <14 days as (0). Note: In Sweden, the first 14 days of sick leave are paid by the employer and thereafter by the national insurance agency.

- How satisfied are you with your weight? The answers 'not at all satisfied' and 'not very satisfied' were dichotomised as dissatisfied (1), and 'neither dissatisfied nor satisfied',' satisfied' and 'very satisfied' as satisfied (0).

- How stressed do you feel? The answers 'very much stress' were dichotomized as (1) and 'little stress', 'just the right level of stress', 'no stress' and 'too little stress' were dichotomized as (0).

- How do you rate your physical health? The answers 'poor' and 'quite poor' were dichotomised as poor (1), and 'excellent', 'very good', and 'good' were dichotomized as good (0).

-How satisfied are you with your present family situation? The answers 'not satisfied at all' and 'not very satisfied' were dichotomized as dissatisfied (1), and 'neither dissatisfied nor satisfied', 'satisfied' and 'very satisfied' as satisfied (0).

-How satisfied are you with your housing situation? Answers 'not at all satisfied' and 'not so satisfied' were dichotomized as dissatisfied (1), and 'neither dissatisfied nor satisfied', 'satisfied' and 'very satisfied' as satisfied (0).

-Do you smoke daily? The answer 'Yes' was dichotomised as (1) and 'no' as (0).

-How much alcohol do you consume during an average week? A picture showing common drinks presenting one standard glass (stg) was shown where e.g. one glass of wine was equal to one stg [25]. The use of ≥ 15 stg in men and ≥ 10 stg in women was dichotomized as (1), and < 15 stg in men and < 10 stg in women as (0).

Patients, who worked or studied, responded also the question:

-How satisfied are you with your working situation? The answers 'not satisfied at all' and 'not so satisfied' were dichotomized as dissatisfied (1) and 'neither dissatisfied nor satisfied', 'very satisfied' as satisfied (0).

### Procedure and inclusion criteria

Our aim was to identify a group of men and women with depression and a reference group of men and women without, among patients who attended the drop-in clinic. Patients were recruited between 1997 and 2001. All patients aged 18-75 years were invited to participate by the two nurses assisting in the study at random week-days. Upon consent, the patients completed the BDI and SC and returned them to the nurse before consultation. Since the prevalence of depression has been shown to be low among men attending primary care [19] we used also a targeted screening approach to avoid screening of an unnecessary large number of patients. Targeted screening is a more realistic approach to use in primary health care, as screening of every patient in primary care is expensive and time-consuming [26]. Patients attending the drop-in clinic, who reported tiredness, anxiety, nervousness, feelings of stress, sleeping or concentration problems were recruited by the two physicians to participate on days the nurses were not available. These patients filled in the BDI and SC after consultation and returned them by post.

The BDI scores were evaluated by the physicians after the consultation and patients with BDI ≥ 10 were invited to a repeat visit within two weeks. At the repeat visit the DSM- criteria were applied to diagnose depression and the severity of depression was assessed with MADRS. Patients' answers in the questionnaires and their health problems were discussed further.

Comparison between the groups of depressed men and women invited by nurses and by the physicians in all explanatory variables in SC and in severity of depression were made with no significant differences found between them. Therefore, the patients from both groups were pooled together to form a single group of depressed men and a single group of depressed women.

Patients with other psychiatric diagnoses than depression and patients who could not answer the questionnaires unaided, e.g. due to dementia or language barrier were excluded.

### Variables

The outcome variable was presence or absence of depression (DSM-IV).

The explanatory variables were:

-Socio-demographic data: Age in years, marital status (single or married/cohabiting), with or without children, high (≥ 12 twelve years) or low (< 12 years) educational background and employment status (student, employed, retired, disability pension and unemployed) (Table 1).

**Table 1:** Mean age with 95% confidence intervals (95% CI) and distribution (%) of socio-demographic data in the 404 participants (48 depressed men and 76 depressed women and 187 men and 93 women without depression).

	Depressed		Non-depressed	
	Men	Women	Men	Women
N	48	76	187	93
*Age*				
Mean	45.6	47.9	44.2	47.7
95% CI	(41.8- 49.4)	(44.5-51.3)	(41.9-46.5)	(44.6-50.9)
*Civil status*				
Living single (%)	55.3	48.7	40.8	38.0
Children, yes (%)	55.6	66.7	46.5	62.4
*Education*				
Secondary school/University (%)	80.1	69.7	82.1	72.3
Employment				
Employed/student (%)	75.0	73.7	80.0	74.2
Unemployed (%)	12.5^1^	6.6	2.2^1^	2.2
Pensioners				
Disability pension (%)	0	4.0	1.6	4.3
Old age pension (%)	12.5	15.8	16.2	19.4

^1 ^Unemployment was more common among depressed men than non-depressed men (p < 0.01, Fisher's test).

-Psychosocial stressors such as poor finances, perceived stress and physical health status as well as dissatisfaction with one's weight, family, housing and work situations (applicable only to participant who worked or studied) (Table 2 and 3).

**Table 2:** Distribution (%) of the self-rated psychosocial stressors and lifestyle choices in depressed *men *versu*s men *without depression.

	Depressed	Non-depressed	P value
N	48	187	
*Psychosocial stressors*	%	%	
Poor finances	31.3	10.8	<0.001
Sick-listed ≥2 weeks past year	35.0	13.9	<0.01
Not satisfied with weight	45.8	34.1	ns
Feeling very stressed	68.8	16.2	<0.001
Poor physical health	68.1	27.2	<0.001
Not satisfied with family situation	31.9	2.2	<0.001
Not satisfied with housing situation	19.2	5.4	<0.01
			
*Life style choices*			
Smoking (yes)	41.7	14.9	<0.001
Alcohol use ≥ 15 standard glasses/week	14.6	10.5	ns
			
*Sub-sample*			
*Employed or studying (n)*	31	135	
	%	%	
Not satisfied with working situation	41.9	5.2	<0.001

*P*-values for tests of differences between the groups.

**Table 3:** Distribution (%) of the self-rated psychosocial stressors and lifestyle choices in depressed *women *versu*s women *without depression.

	Depressed	Non-depressed	P value
N	76	93	
*Psychosocial stressors*	%	%	
Poor finances	32.9	7.5	<0.001
Sick-listed ≥2 weeks past year	23.7	9.7	<0.05
Not satisfied with weight	53.9	33.3	<0.01
Feeling very stressed	63.2	22.6	<0.001
Poor physical health	71.2	41.8	<0.001
Not satisfied with family situation	40.5	4.3	<0.001
Not satisfied with housing situation	22.4	2.2	<0.001
			
*Lifestyle choices*			
Smoker (yes)	16.0	11.0	ns
Alcohol use ≥ 10 standard glasses/week	12.5	4.5	ns
			
*Sub-sample*			
*Employed or studying (n)*	51.0	66.0	
	%	%	
Not satisfied with working situation	33.3	1.5	<0.001

*P*-values for tests of differences between the groups.

- Lifestyle choice questions including tobacco and alcohol use (Table 2 and 3).

### Statistical methods

Differences between groups in categorical variables were tested with Pearson's Chi- square test or Fisher's test. The significance level was set at 5%. Differences between groups of continuous variables are shown as differences in means with 95% confidence intervals (95% CI).

Age adjusted logistic regression as being depressed was used to estimate the odds ratios (OR) with 95% CIs for the explanatory variables, for men and women separately.

Multiple age-adjusted logistic regression analyses were performed with manual stepwise forward variable selection. Model improvement was tested by applying the likelihood ratio test where p < 0.05 indicated a model improvement. The Hosmer-Lemeshow test was used to assess the goodness of fit of the model and was considered as satisfactory if p was > 0.05.

Differences in Beta coefficients between men and women in main effect models were calculated as the differences between Beta-coefficients divided by the square root of the sum of the squared standard errors for each variable. If this ratio was larger than 1.96 the difference was considered statistically significant.

Values for receiver operating curves (ROC) for the age-adjusted main effect models are also shown. A value above 0.8 was considered to represent a good distinction.

The positive and negative predictive values of the main effect models were analysed separately for men and women.

Statistical analyses were performed using the software package of STATA 8 [27].

### Ethical Approval

Approval was obtained from The Ethics Committee of Huddinge Hospital, Sweden.

## Results

### The study group

In total, 513 patients were invited to participate. Of the 299 men, a total of 266 were invited by the nurses and 33 by the physicians. The participation rate in men was 82.6%. The men who declined were significantly older. Of the 74 men invited to the interview, (BDI ≥10) twelve men (16.2%) abstained and they did not differ significantly from the men who were interviewed. Of the 214 invited women, 155 were invited by the nurses and 59 by the physicians. The participation rate in women was 83.2%. The women who declined did not significantly differ in age from the ones interviewed. Of the 98 women invited to the interview (BDI ≥10) nine women (9.2%) abstained. The abstainers were significantly younger than the interviewed women.

Taking all above into account, the total study group consisted of 404 participants: The 48 depressed men (nurse-invited n = 26, physician invited n = 20), the 76 depressed women (nurse-invited n = 35, physician invited n = 41) and the reference groups of 187 men and 93 women. The reference group consisted both of the patients with BDI < 10 and those with BDI ≥ 10 who had no psychiatric diagnosis. One third of the depressed men and half of the depressed women had depression of moderate severity according to MADRS and the remainder had mild depression.

### Socio demographic data

The majorities of the participants were in their forties and had education at university level (Table 1). About half were single and one third had no children. There were no significant differences between the depressed and the reference groups in the socio demographic variables, except for unemployment, which appeared significantly more common in the depressed men compared to the men without depression in the sub-sample who worked or studied (p < 0.01).

### Psychosocial stressors and lifestyle questions in men and women

Table 2 shows the distribution (%) of the self-rated psychosocial stressors and lifestyle choices in depressed versus non-depressed men. All, except two variables (not feeling satisfied with weight and alcohol use ≥ 15 stg) were significantly more often reported by the depressed men than non-depressed men.

Table 3 shows the corresponding data for women. All psychosocial stressors, except for daily smoking and alcohol use >10 stg, were significantly more often reported by the depressed women than by non-depressed women.

Notably, comparing Table 2 and Table 3 there was a difference between men and women. Smoking was significantly more common only in depressed but not in depressed women and dissatisfaction with weight was only significantly more common in depressed women but not in men.

In the sub-samples of employed participants depressed men and women reported dissatisfaction with their work situations significantly more often than men and women without depression (p < 0.001).

### Odds ratios for the explanatory variables, main effect models and positive and negative predictive values in men and women

The significant variables from Tables 2 and 3 were included as explanatory variables in the logistic regression. Table 4 and 5 show the age-adjusted ORs with 95% CI for the explanatory variables as predicting depression in men and women, respectively. In both men and women, all explanatory variables had significant ORs. The same three variables constituted the main effect model in men and women, which included "feeling very stressed", "perceiving poor physical health" and "not being satisfied with one's family situation". The ROC values for the main effect models were 0.88 in men and 0.83 in women. No statistically significant differences concerning the separate variables in the age-adjusted main effect models in men and women were found.

**Table 4:** Age-adjusted odds ratios (OR) with 95% confidence intervals (95% CI) for risk-factors associated with depression in *men *and in the age-adjusted main effect model.

Variable		Main effect model
	**OR (95%CI)**	**OR (95%CI)**

Poor finances	3.7 (1.7 - 8.0)	

Unemployed	3.6 (1.1 - 11.4)	

Sick-listed ≥ 2 weeks past year	3.4 (1.7 - 7.1)	

Feeling very stressed	11.9 (5.7 - 24.1)	10.4 (4.3 - 25.1)

Poor physical health	5.8 (2.7 - 11.6)	3.6 (1.5 - 8.5)

Not satisfied with family situation	20.9 (6.5 - 67.4)	22.4 (5.8 - 86.8)

Not satisfied with housing	4.3 (1.6 - 11.5)	

Smoker	3.8 (1.6 - 8.8)	

Hosmer-Lemeshow goodness of fit value = 0.52, ROC value = 0.88 of the main effect model.

**Table 5:** Age adjusted odds ratios (OR) with 95% confidence intervals (95% CI) for risk-factors associated with depression in *women *and in the age-adjusted main effect model.

Variable	Age-adjusted	Main effect
	**OR (95%CI)**	**OR (95%CI)**

Poor finances	6.95 (2.7 - 17.9)	

Sick-listed ≥ 2 weeks past year	2.9 (1.2 - 6.9)	

Feeling very stressed	5.9 (3.0 - 11.6)	5.1 (2.4 - 11.1)

Poor physical health	3.6 (1.8 - 6.9)	3.1 (1.4 - 6.6)

Not satisfied with family situation	16.1 (5.3 - 49.1)	13.6 (4.0 - 46.2)

Not satisfied with housing situation	15.5 (3.3 - 71.9)	

Hosmer - Lemeshow goodness of fit value = 0.77, ROC values = 0.83 of the main effect model.

Finally, the positive predictive values for depression of the main effect models with the three explanatory variables were 64.4% in men and 78.7% in women. The negative predictive value for depression in men was 90.7% and 76.5% in women.

Separate age-adjusted logistic regressions were performed concerning the variable being dissatisfied with one's working situation for men and women who worked. The ORs for depression were 13.2 (95% CI 4.7 - 37.5) in men and 32.5 (95% CI 4.1 - 254.7) in women.

## Discussion

In this study, we found that the same three psychosocial stressors: "being dissatisfied with one's family situation", "feeling very stressed" "and "perceiving poor physical health" were equally associated with depression in men and women. Moreover, "dissatisfaction with one's working situation" was associated with depression in men and women. However, unemployment and daily smoking were associated with depression only in men, whereas dissatisfaction with weight was associated with depression only in women.

The study showed that questions concerning psychosocial stressors are relevant for physicians to ask, even when patients are well established in Swedish society. Presence of these stressors can be used to identify patients at risk for depression, who could be invited to a follow up. This idea is supported by a recent Danish study, which showed that a screening strategy based on physicians' clinical eye coupled with a short validation test was effective in detection of depression [28]. This is important as routine screening of patients for depression is not cost-effective [26].

The positive predictive value of the main effect model was higher in women than in men. In addition, the positive predictive value of 64.6% we observed in women was higher than that for family physicians who diagnosed depression without any screening instrument and was reported to be 34% [29].

The negative predictive value of the main effect model was higher in men than in women. A negative predictive value for a common clinical condition is an important tool for physicians in primary health care. In practice, this observation would mean that men, who do not report stress, consider themselves as physically healthy and are satisfied with their family situations are at low risk of depression. On the other hand, the negative predictive value in women was lower and, therefore, of uncertain clinical value. The negative predictive value for family physicians' unaided clinical diagnosis (without using any instruments) was reported to be 91% [29].

The answers in the SC did not directly point out what circumstances the participants refer to when, for example, rating their family situation as poor. However, the discussion at the repeat visit revealed problems in close relationships, both to close and more distant relatives as well as to ex-spouses. Problems could include human relations, worries because of an illness of a family member, or perceived stress when caring for elderly parents.

Psychosocial stressors are common especially in people with low educational background, blue collar workers and immigrants [30]. However, our study may imply that such stressors are not limited only to those groups. Even our relatively affluent group reported psychosocial stressors associated with depression such as job cutbacks, especially in the public sector, where many of the women worked. To note, this study showed that men and women in our study group reported the same three main psychosocial stressors equally. This may reflect recent changes in gender roles in Sweden, where men take a more active part in household activities and child care and women pursue career more often than before.

Overall, our observations imply that more resources are needed in primary health care to help depressed men and women cope with psychosocial stressors. At the beginning of the study, patients with depression were commonly referred to social workers in primary care at a low cost, as psychological treatment was considered too expensive by many patients. Today, psychological treatments with a limited number of psychologist consultations are subsidised by the Stockholm County Council. Moreover, the guidelines for management of depression in Swedish primary health care currently recommend cognitive behavioural therapy as psychological treatment for depression.

### Strengths and limitations

This study is one of the first in Swedish primary care covering the aspects psychosocial stress and depression. The major strength is the close connection to every day practice in primary care. In addition, strict diagnostic criteria for depression using DSM-IV were applied and the severity of depression was reported. Limitations were mainly the setting and cross-sectional design. Specifically, all participants recruited lived in an area of higher socio-economic status (SES) than average and, therefore, the results could only be indicative of patient populations with similar status. Another limitation is that data were collected ten years ago. However, human reactions to stressors do not change rapidly and, therefore; we consider our results relevant to the today's reality. Finally, the cross-sectional design made us unable to infer any causal association between psychosocial stressors and depression.

### Comparisons with other studies

Our study differs from many other studies as it includes a relatively affluent study population. A number of socio-economic, psychosocial, cultural and gender related factors have been identified as risk factors for depression in population studies, including female gender, low SES, financial stress, unemployment, work stress, social isolation and poor housing situation [11,31,32]. A study from primary health care in Switzerland showed a stronger association between psychosocial stressors and depression, anxiety and somatoform disorders than that between socio-demographic determinants (educational level, occupation, marital status) and these mental disorders [33]. Our results are consistent with these findings. In a large multinational study a risk algoritm for predicting depression in primary care was developed, including socio-demographic factors but also discrimination, physical ill-health and family history of psychological difficulties [34]. A Finnish primary care study reported that the depressed men had lower social functioning compared to women and pointed out a need to pay special attention to the needs of depressed men [35]. Results from one qualitative study are also of interest, where the depressed patients believed that their depression was caused by current life stressors and personal characteristics [36]. In our study, unemployed women were less likely to be depressed than unemployed men, just as in the Hampshire Depression Project [37]. Women might be more resistant to the stress of unemployment, as men and women have may different life priorities [38]. In addition, a Finnish primary care study found similar risk factors for depression in men and women to those we identified here, which suggests that marital and interpersonal relationships are important issues for physicians to discuss with their patients [16].

### Clinical implications

All patients hade depressions of mild or moderate severity and many depressed men and women reported problems with their family and work situations and were feeling stressed. Thus, they were suitable for treatment in primary health care, indicating that resources for psychosocial support should be allocated to primary health care. Integrating key questions about psychosocial stressors in a routine consultation would be a useful tool to identify patients at high risk of depression and improve early detection of depression.

## Conclusions

Gender differences were restricted to unemployment, smoking and weight dissatisfaction. The same three psychosocial stressors were found to be associated with depression equally in men and women. Thus, questions often asked by physicians about family relations, stress, perceived physical health and work situation are relevant indicators in detection of patients at risk of depression.

## Competing interests

The authors declare that they have no competing interests.

## Authors' contributions

The study was designed in cooperation with RS, Professor Anna-Åberg-Wistedt at the Department of Clinical Neuroscience, Section of Psychiatry and psychiatrist at the Affective Centre at St Göran's Hospital, Stockholm and MD Estera Wernering at the Serafen Health Care Center, Stockholm. RS carried out data collection and the statistical analyses. The literature survey was made by RS in cooperation with ML. The manuscript was drafted by RS and ML and developed through discussion with LGB. All authors approved the final manuscript.

## Authors' information

RS is an MD, is general practitioner at the Serafen Health Care Center, Stockholm. LGB is an MD, PHD and general practitioner at the Gustavsberg Health Care Center, Stockholm, ML, is an MD, Assistant Professor and general practitioner at Tisken Health Care Center, Falun, Sweden.

## Pre-publication history

The pre-publication history for this paper can be accessed here:

http://www.biomedcentral.com/1471-2296/12/120/prepub
